# Binding of *Shewanella* FadR to the *fabA* fatty acid biosynthetic gene: implications for contraction of the *fad* regulon

**DOI:** 10.1007/s13238-015-0172-2

**Published:** 2015-06-07

**Authors:** Huimin Zhang, Beiwen Zheng, Rongsui Gao, Youjun Feng

**Affiliations:** Department of Medical Microbiology & Parasitology, Zhejiang University School of Medicine, Hangzhou, 310058 China; State Key Laboratory for Diagnosis and Treatment of Infectious Diseases, Zhejiang University School of Medicine, Hangzhou, 310058 China

**Keywords:** FadR, *fad* regulon, *fabA*, *fabB*, contraction, *Shewanella*

## Abstract

The *Escherichia colifadR* protein product, a paradigm/prototypical FadR regulator, positively regulates *fabA* and *fabB*, the two critical genes for unsaturated fatty acid (UFA) biosynthesis. However the scenario in the other Ɣ–proteobacteria, such as *Shewanella* with the marine origin, is unusual in that Rodionov and coworkers predicted that only *fabA* (not *fabB*) has a binding site for FadR protein. It raised the possibility of *fad* regulon contraction. Here we report that this is the case. Sequence alignment of the FadR homologs revealed that the N-terminal DNA-binding domain exhibited remarkable similarity, whereas the ligand-accepting motif at C-terminus is relatively-less conserved. The FadR homologue of *S. oneidensis* (referred to FadR_she) was over-expressed and purified to homogeneity. Integrative evidence obtained by FPLC (fast protein liquid chromatography) and chemical cross-linking analyses elucidated that FadR_she protein can dimerize in solution, whose identity was determined by MALDI-TOF-MS. *In vitro* data from electrophoretic mobility shift assays suggested that FadR_she is almost functionally-exchangeable/equivalent to *E. coli* FadR (FadR_ec) in the ability of binding the *E. coli fabA* (and *fabB*) promoters. In an agreement with that of *E. coli fabA*, *S. oneidensisfabA* promoter bound both FadR_she and FadR_ec, and was disassociated specifically with the FadR regulatory protein upon the addition of long-chain acyl-CoA thioesters. To monitor *in vivo* effect exerted by FadR on *Shewanella fabA* expression, the native promoter of *S. oneidensisfabA* was fused to a LacZ reporter gene to engineer a chromosome *fabA-lacZ* transcriptional fusion in *E. coli*. As anticipated, the removal of *fadR* gene gave about 2-fold decrement of *ShewanellafabA* expression by β-gal activity, which is almost identical to the inhibitory level by the addition of oleate. Therefore, we concluded that *fabA* is contracted to be the only one member of *fad* regulon in the context of fatty acid synthesis in the marine bacteria *Shewanella* genus.

**Electronic supplementary material:**

The online version of this article (doi:10.1007/s13238-015-0172-2) contains supplementary material, which is available to authorized users.

## INTRODUCTION

Current knowledge on the regulation of fatty acid metabolism is mostly from studies with *Escherichia coli* (*E. coli*). The *E. coli* FadR regulatory protein that is classified into the GntR family of transcription factors, acts as a global regulator controlling bacterial lipid metabolism (Henry & Cronan, 1992, Iram & Cronan, 2005). The two opposite roles played by this regulator include repression of fatty acid degradation (*fad*) system (Feng & Cronan, 2009b, Henry & Cronan, 1991, Iram & Cronan, 2005), and activation of *fabA* and *fabB*, the two genes for unsaturated fatty acid synthesis (Feng & Cronan, 2009a, Henry & Cronan, 1992, Nunn et al., 1983). In fact, the *E. coli* FadR also indirectly regulates transcription of the glyoxylate bypass operon (*aceBAK*), through direct activating the IclR repressor (Gui et al., 1996). Very recently, My et al. (My et al., 2013) reported that *E. colifabH* is the third gene of fatty acid synthesis pathway that can be positively-regulated by the FadR regulator.

As the paradigm FadR regulator, the *E. colifadR* protein product behaves as a dimer (van Aalten et al., 2000), and consists of the N-terminal DNA-binding domain (Xu et al., 2001) and the ligand-interacting motif at C-terminus (van Aalten et al., 2001). The accumulated crystal structures of FadR alone and its complex with DNA/acyl-CoA defined clearly the structural basis for FadR-mediated regulatory mechanism in the context of lipid metabolism (van Aalten et al., 2001, van Aalten et al., 2000, Xu et al., 2001). In addition to the residues directly contacting target DNA (Xu et al., 2001), we also mapped three more key residues with indirect role in FadR-DNA interplay (Zhang et al., 2014). *In vitro* and *in vivo* evidence proved that long-chain fatty acid (LCFA) acyl-CoA thioesters are small molecule effectors for the FadR regulatory system (Henry & Cronan, 1992, van Aalten et al., 2001, Cronan, 1997). The mechanism by which LCFA induces *fad* expression lies in the fact that the binding of LCFA acyl-CoA to FadR protein results in the alteration of protein configuration, which in turn triggers the loss of its DNA binding ability. However, it still remains unclear why the unexpected functional diversity exists amongst the FadR regulatory proteins (Iram & Cronan, 2005). Of particular note, it is mystery that in relative to the prototypical FadR with an origin of *E. coli*, the *Vibrio cholerae* (*V. cholerae*) FadR is strikingly superior to in the regulatory amplitude, and bound its ligands appreciably stronger (Iram & Cronan, 2005). Further sequence analyses revealed that an extra 40-aa longer region present in *V. cholerae* FadR might explain the excellent performance of its regulation role in the context of lipid metabolism (Zhang et al., 2014). Unlike the scenario seen with its closely-relative *V. cholerae*, the FadR homologue from the other marine bacterium *Shewanella*, is quite similar to that of the paradigm organism *E. coli*.

The genus of *Shewanella* is a family of Gram-negative bacteria inhabiting in marine environment/ecosystem, including no less than 50 diversified species such as *S. oneidensis* and *S. algae* (Janda & Abbott, 2014). *S. oneidensis* is referred to an alternative model anaerobic micro-organism with the known genome sequence (~4.9 Mb) that encodes over 4700 genes (Kolker et al., 2005, Heidelberg et al., 2002). Not only do the species of *Shewanella* bacteria (e.g., *S.putrefaciens*) act as normal components of the surface flora of fish and are involved in the spoilage of aquatic products (Parlapani et al., 2013, Li et al., 2012), but also some species like *S. algae* is recognized to be zoonotic pathogens in that they can cause opportunistic infections via occupational exposure of workers with skin and soft tissue cuts to marine products (Janda & Abbott, 2014). Given the excellent performance of *S. oneidensis* in reduction of poisonous heavy metals like iron (Cheng et al., 2013), uranium (Sheng & Fein, 2014), and even ionic mercury (Wiatrowski et al., 2006), it was believed to have the robust/potential applications into environmental bioremediation targeting toxic elements and heavy metals and development of microbial fuel cells (Fredrickson et al., 2008, Hau & Gralnick, 2007). The advantage of *Shewanella* in biotechnology is mainly attributed to the diversified metabolic capabilities that included versatile electron-transfer systems (Hau & Gralnick, 2007, Fredrickson et al., 2008). The deep-sea environment with low temperature where the *Shewanella* bacteria naturally reside/inhabit determined that some special mechanism might be evolved for their survival. Wang et al. (Wang et al., 2009) found that *Shewanella* has appreciable ability to produce various types of low-melting-point fatty acids with monounsaturated fatty acids (MUFA) included. The similar scenario was also noted in the other marine bacterium *V. cholerae*, in which relatively-high percentage of unsaturated fatty acids (UFA) is present in relative to *E. coli* (Massengo-Tiasse & Cronan, 2008, Feng & Cronan, 2011a). The physiological explanation proposed lies in that the high percentage of UFA in the bacterial membrane incorporated with phospholipid confers the better membrane fluidity, which in turn enhances its capability of cold adaptation. Although the type II fatty acid synthesis (FAS) pathway in *Shewanella* was constructed using the approach of comparative genomics (Wang et al., 2009), it seemed likely that some unusual/unclear aspects are present in the regulation of this specialized Type II FAS (Rodionov et al., 2011). Of particular note, Rodionov and coworkers (Rodionov et al., 2011) predicted that only *fabA* (not *fabB*) of *Shewanella* has a binding site for FadR protein, posing the possibility of *fad* regulon contraction.

In this paper, we integrated *in vitro* and *in vivo* approaches to address this uncommon question and reported that this is the case. As expected, *Shewanella* FadR regulates expression of *fabA* (not *fabB*) through the direct protein-DNA physical interplay. Therefore, it is reasonable that *the fabA* fatty acid synthesis gene is contracted as the only one member of *fad* regulon in the context of fatty acid synthesis in the marine bacteria *Shewanella* genus.

## RESULTS AND DISCUSSION

### Contraction of *fad* regulon in *Shewanella*

Different from the paradigm enteric bacterium *E. coli* that has only one chromosome of 4.64 Mb with average GC contents of, 50.8% and encodes 4498 putative genes (Blattner et al., 1997), *S. oneidensis* MR-1, the representative cousin with marine origin, not only has a chromosome (4.96 Mb, 46% GC percentage) encoding 4403 genes, but also contains a mega-plasmid (0.16 Mb, 43.7% GC percentage) encoding 149 putative proteins (Heidelberg et al., 2002, Kolker et al., 2005). The similar scenario was also seen with *V. cholerae* N16961, its closely-relative with the same marine origin, in that it carries two genomes (one of which is 2.98 Mb with 47.7% GC contents and encodes 2690 genes, and other one is 1.07 Mb (46.9% GC percentage) corresponding to 1003 genes (Heidelberg et al., 2000).

**Figure 1:**
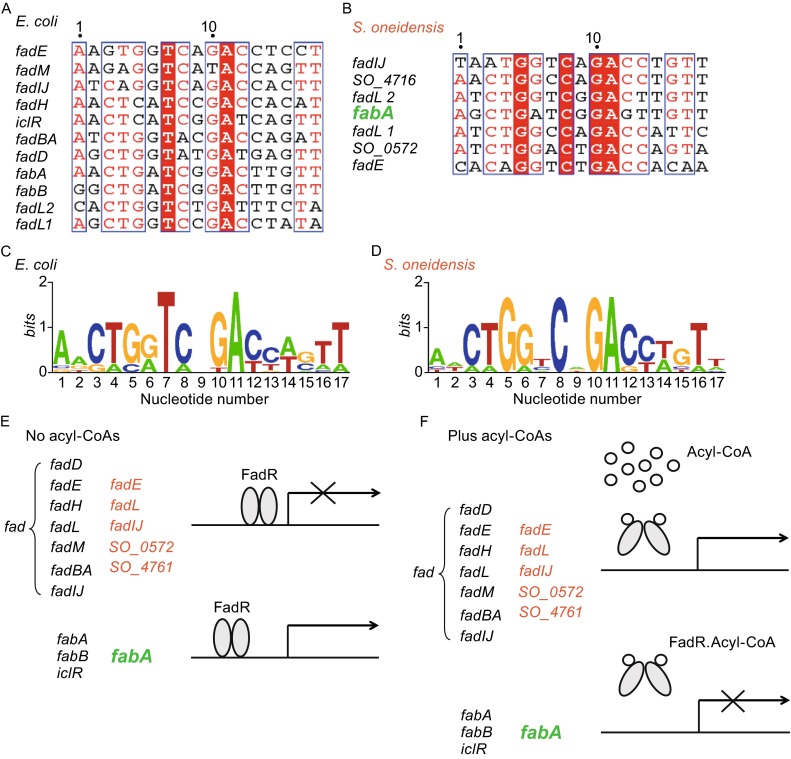
**The working model proposed for** ***fad*** **regulon and its regulation in** ***Shewanella*** **genus**. Multiple sequence alignments (A) and sequence logo (C) of the known palindromes recognized by *E. coli* FadR. Sequence analyses (B) and sequence logo (D) of the predicted FadR-binding sites of *Shewanella*. The alignment of DNA sequences was carried out using ClustalW2 (http://www.ebi.ac.uk/Tools/clustalw2/index.html), and the output was given via processing with the program ESPript 2.2. (http://espript.ibcp.fr/ESPript/cgi-bin/ESPript.cgi) (Feng & Cronan, 2011b). Identical residues are in white letters with red background, similar residues are in the form with mixture of red/black letters, and the varied residues are in black letters. Sequence logo of the FadR-binding sites was generated using the program of WebLogo (http://weblogo.berkeley.edu/logo.cgi). The sequences of the known *E. coli* FadR sites were sampled from *E. coli* K-12 MG1655 (http://regprecise.lbl.gov/RegPrecise/regulon.jsp?regulon_id=10286), and the putative *Shewanella* FadR sites were collected from *S. oneidensis* MR-1(http://regprecise.lbl.gov/RegPrecise/regulon.jsp?regulon_id=5431). (E) In the absence of a long chain acyl-CoA, *E. coli* FadR of *E. coli* and *S. oneidensis* represses the *fad* regulon genes, whereas it activates transcription of *fabA* (and/or *fabB*) with critical roles in the unsaturated fatty acid synthetic pathway. (F) Binding of long chain acyl-CoA species leads to the release of FadR protein from its operator sites. The *fad* members of *Shewanella* are in red except that *fabA* is highlighted in green. Such kind of FadR-DNA dissociation increases *fad* regulon expression whereas reduces the expression of *fabA* (and/or *fabB*). The oval denotes FadR regulatory protein whereas the small open circle represents the acyl-CoA pool

In relative to *E. coli* that has no less than 12 *fad* members, those genes controlled by the fatty acid-responsive FadR regulator (Fig. 1A, 1C and 1E), the *fad* members seemed to be contracted in the cousin *S. oneidensis* in that only 4 well-known *fad* genes/operons (*fadE*, *fadL*, *fadIJ* and *fabA*) has the putative FadR-binding sites (Fig. 1B, 1D and 1F). Also, *S. oneidensis* possesses two more new FadR-regulated genes (SO_4761 encoding the GNAT family of Acetyltransferease and SO_0572 representing a possible Enoyl-CoA hydratase (EC 4.2.1.17)) (Fig. 1B and 1F), somewhat suggesting the expansion of limited *fad* members in this marine bacterium. However, this observation argues the possibility of gene horizontal transfer in that the GC contents of two genes SO_4761 (45.23%) and SO_0572 (46.84%) is similar to that of the whole genome (46%). Unlike *E. coli* that encodes only one FadL fatty acid transporter (Blattner et al., 1997), *S. oneidensis* has three FadL-like homologues (FadL-1 (SO_3099, 440 aa), FadL-2 (SO_3276, 311 aa) and FadL-3 (SO_4232, 437 aa)) (Heidelberg et al., 2002), only FadL-1 of which is directly regulated by FadR regulatory protein (Fig. 1B and 1F). This situation seems unusual, but not without any precedent. The similar scenario was observed in *V. cholerae*, the other marine bacterium since three FadL orthologues are distributed in its two chromosomes (Heidelberg et al., 2000), and only FadL-2 is regulated by FadR repressor (http://regprecise.lbl.gov/RegPrecise/regulon.jsp?regulon_id=16367). It is reasonable that three FadL transporters coupled with one FadD inner-membrane protein (3FadL-1FadD) constitute a more efficient system of fatty acid uptake than the prototypical version of 1FadL-1FadD in *E. coli*. Unlike the *V. cholerae* FadL-2 that has only one FadR-recognizable site not shown), *S. oneidensis* FadL-1 exhibits two tandem FadR-binding sites (Fig. 1B), similar to the scenario seen in *E. coli* FadL (Fig. 1A). Different from the *E. colifadD* that also carries two tandem FadR-specific palindromes, the *fadD* gene with origins of both *S. oneidensis* and *V. cholerae* seems not to be regulated by the FadR regulator in that the typical site is cryptic (Fig. 1B). In views of genomic evolutions, we anticipated that *S. oneidensis* has the relics of both *E. coli* and *Vibrio* in the context of fatty acid transporter system.

Given the significant difference of their inhabiting environments/ecological niches (*E. coli* is enteric bacterium living in fatty acids-rich gastro-intestine, whereas *Vibrio* and *Shewanella* both inhabit in the environment of fresh/salt water with poor fatty acids (Giles et al., 2011).), we believed that this kind of fatty acid uptake system might represent an evolutional/physiological advantage for these marine bacteria to scavenge the limited availability of exogenous fatty acids. Intriguingly, comparative analyses of the GC percentage, an indicator of gene horizontal transfer, showed that FadL-2 (48.61%) is appreciably lower than the average value of the whole genome (46%), implying it might be obtained by gene horizontal transfer, whereas FadL-1 (45.43%) and FadL-3 (46.73%) not (not shown).

In generally consistence with the earlier observations with *V. cholerae* (Feng & Cronan, 2011a, Rodionov et al., 2011), we noted that only the *fabA* gene from the FabA-FabB UFA synthesis pathway in *Shewanella* has a known FadR-binding site (Figs. 1B and S1A), whereas the *fabB* gene does not (Fig. S1B). It suggested the presence of an asymmetric/unparalleled FadR regulation in *Shewanella* (Fig. 1E and 1F). these findings might argue the conclusions by Shi and coauthors (Shi et al., 2015) in the case of the regulated-expression of the *fabB* gene in its closely-relative, *V. cholerae*, in that only indirect role of FadR can be assigned due to the absence of the FadR-binding site. Our observations plus the predictions by Rodionov et al. (Rodionov et al., 2011), supported the proposal that the *fad* regulon contraction is present in *Shewanella*. Given the important physiological role of the *fabA* gene in bacterial UFA synthesis, we attempted to experimentally verify this unusual hypothesis.

### Characterization of *Shewanella* FadR

**Figure 2:**
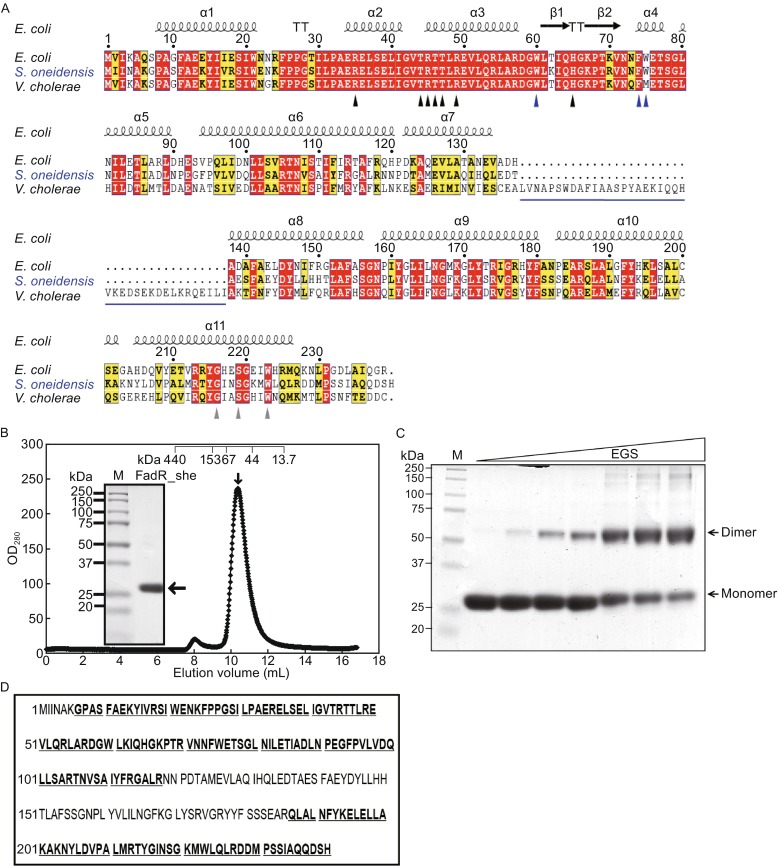
**Characterization of** ***S. oneidensis*** **FadR protein**. (A) Sequence analyses of three different FadR homologues. The multiple alignments of FadR protein sequences were performed using ClustalW2 (http://www.ebi.ac.uk/Tools/clustalw2/index.html), and the resultant output was processed by program ESPript 2.2 (http://espript.ibcp.fr/ESPript/cgi-bin/ESPript.cgi), generating the final BLAST photography (Feng & Cronan, 2011b). Identical residues are in white letters with red background, similar residues are in black letters in yellow background, the varied residues are in grey letters, and gaps are denoted with dots. In light of the structural architecture of *E. coli FadR* protein (PDB:1E2X) (van Aalten et al., 2000), the protein secondary structure was illustrated in cartoon (on top) (Zhang et al., 2014), α: alpha-helix; β: beta-sheet; T: Turn; η: coil. The seven known DNA-binding sites (R35, T44, R45, T46, T47, R49 and 65H) are highlighted with black triangles (Xu et al., 2001), the three known ligand-binding sites are shown with grey triangles (216G, 219S and 223W) (van Aalten et al., 2001), and the newly-proposed amino acids with indirect role for FadR-DNA interaction are highlighted with blue arrows (W60, F74 and W75) (Zhang et al., 2014). The extra 40-aa (138–177) longer region of *V. cholerae* FadR was underlined in blue. The FadR sequences are separately sampled from *E. coli* K12 (Accession no.: CAA30881), *V. cholerae* (*Vibrio cholerae*) (Accession no.: AAO37924) and *S. oneidensis* (Accession no.: NP_718457). (B) Gel exclusion chromatographic profile of the recombinant *S. oneidensis* FadR protein run on a Superdex 75 column (GE Healthcare). The expected peak of the target FadR was eluted at the position of 10.5 mL (highlighted with an arrow). The inset gel is the 15% SDS-PAGE photography of the collected *S. oneidensis* FadR protein sample. The mass of the monomeric *S. oneidensis* FadR is estimated to be ∼27 kDa. Abbreviations: M, protein marker; OD_280_, optical density at 280 nm; mAu, milli-absorbance units. The ruler on the top was given to describe the elution pattern of the standard proteins (Pharmacia). The standards used here included Ferritin (∼440 kDa), Aldolase (153 kDa), Bovine serum albumin (∼67 kDa), Ovalbumin (∼44 kDa) and ribonuclease (∼13.7 kDa), respectively. (C) Chemical cross-linking analyses for the purified *S. oneidensis* FadR protein. The level of EGS chemical cross-linker was illustrated with a triangle varies from 0, 0.1, 0.2, 0.5, 1.0, 1.5, to 2.0 µmol/L. (D) MS determination of the recombinant *S. oneidensis* FadR protein. The matched amino acid residues that exhibited 69% coverage to the native *S. oneidensis* FadR are given bold and underlined type

An earlier study (Iram & Cronan, 2005) has found that the FadR lipid metabolism regulator of *V. cholerae* has an unusual insert of 40 residues. Our results (submitted) plus Shi’s observations (Shi et al., 2015) revealed an unexpected contribution of this unique inserting sequence in constituting an extra-ligand binding motif for FadR regulatory protein. The second ligand-binding site confers its excellent ability in fatty acid sensing. Given the fact that both *V. cholerae* and *S. oneidensis* are closely-related marine bacteria that shared a similar ecological niche with poor availability of fatty acids, we initially anticipated that this insert might be an indicator or relic for such kind of unparalleled regulation by FadR (Fig. 1). In fact, it is not this case. Multiple sequence alignments of three FadR proteins (FadR_ec for *E. coli*, FadR_vc for *V. cholerae*, and FadR_she for *S. oneidensis*) showed that: 1) the N-terminal DNA-binding motifs are very conserved featuring a full set of all the known residues critical for DNA binding; 2) the C-terminal ligand-interacting domains are appreciably diversified; and 3) the so-called insert of 40 residues (138–177 aa) is only present in FadR_vc (Fig. 2A). Considered the fact combined with atypical features seemed in fatty acid transport system, we favored the anticipation that *Shewanella* somewhat retains the evolutional relic that are partially observed with *E. coli* and *Vibrio*, respectively.

To further functional analyses of the above bacterial FadR proteins, we over-expressed the three types of recombinant FadR proteins (FadR_ec FadR_vc & FadR_she) and purified them to homogeneity. As expected, SDS-PAGE profile clearly showed the purified FadR_she protein migrates at the position of ~27 kDa. The FPLC profile showed that the expected peak of purified *S. oneidensis* FadR was eluted at the position of 10.5 mL (indicated with an arrow, Fig. 2B), suggesting its apparent molecular mass is more than 44 kDa, but less than 67 kDa. Given the fact that the ideal molecular weight of recombinant *S. oneidensis* FadR in momomer is ~27 kDa, we believed that the form of FadR_she in solution might be a dimer (-54 kDa). It was generally consistent with the scenario seen with the *E. coli* FadR as a dimer. Subsequently, we used chemical cross-linking assays to further prove this speculation. As we expected, appearance/formation of the dimerization for the FadR_she protein is appreciably increased upon addition of chemical cross-linker EGS (Fig. 2C). Also, it behaves in an EGS dose-dependent manner (Fig. 2C). In particular, the dimer band was excised from the SDS-PAGE and subjected to liquid chromatography mass spectrometry. As a result, the MS results confirmed this identity in that the digested peptides matched the *S. oneidensis* FadR protein with the coverage of 69% (Fig. 2D).

### The FadR proteins of *E. coli* and *S. oneidensis* are functionally-equivalent

**Figure 3:**
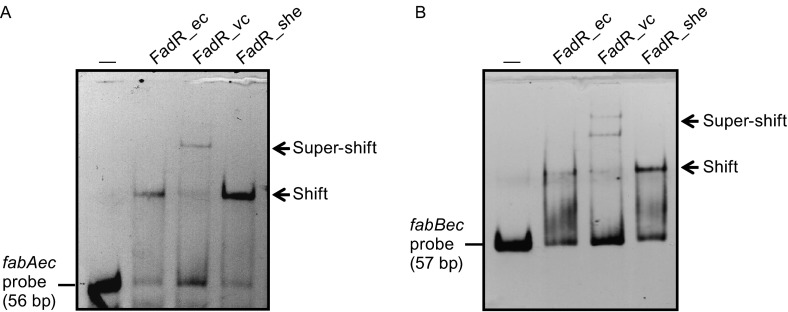
***Shewanella*** **FadR protein is functionally-exchangeable to the paradigm** ***E. coli*** **version**. (A) EMSA-based evidence for binding of *E. coli fabA* promoter to FadR protein of three origins. (B) EMSA analyses for crosstalk of *E. coli fabA* promoter with three kinds of bacterial FadR proteins. A representative photography was given here, which were from no less than three independent EMSA experiments (7% native PAGE). Three versions of FadR protein here are FadR_ec, FadR_vc and FadR_she, respectively. In gel shift assays, the FadR protein (5 pmol) is incubated with DIG-labeled *fabA*ec (or *fabB*ec) probe (0.2 pmol). Note: An unexpected but interesting scenario “super-shift” is consistently observed in our trials with *V. cholerae* FadR

Gel shift assay was performed to detect the binding ability of FadR_ec, FadR_vc and FadR_she to the cognate DNA binding sites. As expected, EMSA-based experiments showed that the *E. coli* FadR protein (as the positive control) binds well to its own promoters of both *fabA* (Fig. 3A) and *fabB* (Fig. 3B) promoters. The fact that the FadR_vc protein gives consistently the super-shift bands for both *fabA* and *fabB* probes in the gel shift assays, is mostly attributed to the essence of its easy-forming the protein multimer (Fig. 3). Of note, FadR_she exhibited an excellent ability of interacting with the *fabA* (Fig. 3A) and *fabB* (Fig. 3B) with the origin of *E. coli*. It seemed likely that the FadR proteins of *E. coli* and *S. oneidensis* are functionally-equivalent (and/or exchangeable). It is not surprise since the FadR/FabR orthologue from other marine bacterium, *Vibrio*, also followed this rule (Feng & Cronan, 2011b, Feng & Cronan, 2011a). To our knowledge, the cases of similar functional exchange of transcriptional regulators can be extended to BioR, the other GntR-type regulators implicated into the metabolism of biotin, a sulfur-containing fatty acid (Feng et al., 2013a, Feng et al., 2013b). Thereby, it makes sense that the atypical regulation by FadR in UFA synthesis of *Shewanella* is due to the cryptic site in front of *fabB* (Fig. 1), not FadR_she (Fig. 3).

### *S. oneidensis fabA* has a functional FadR-binding site, and this binding is specifically reversed by long-chain acyl-CoA

**Figure 4:**
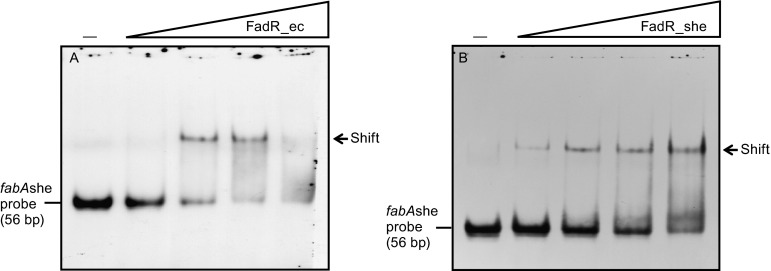
**Evidence that** ***Shewanella*** ***fabA*** **promoter has a functional FadR-recognizable palindrome**. (A) Binding of *Shewanella fabA* promoter to *E. coli* FadR protein. (B) Interplay between *Shewanella fabA* promoter and *Shewanella* FadR protein. The gel shift tests were conducted using 7% native PAGE, and a representative result is shown here. In these assays, levels of FadR protein (FadR_ec and FadR_she) added are denoted with a triangle on right hand (0.1, 0.5, 2, and 5 pmol), whereas the DIG-labeled *fabB*she probe is added to 0.2 pmol. Minus sign denotes no addition of FadR protein

**Figure 5:**
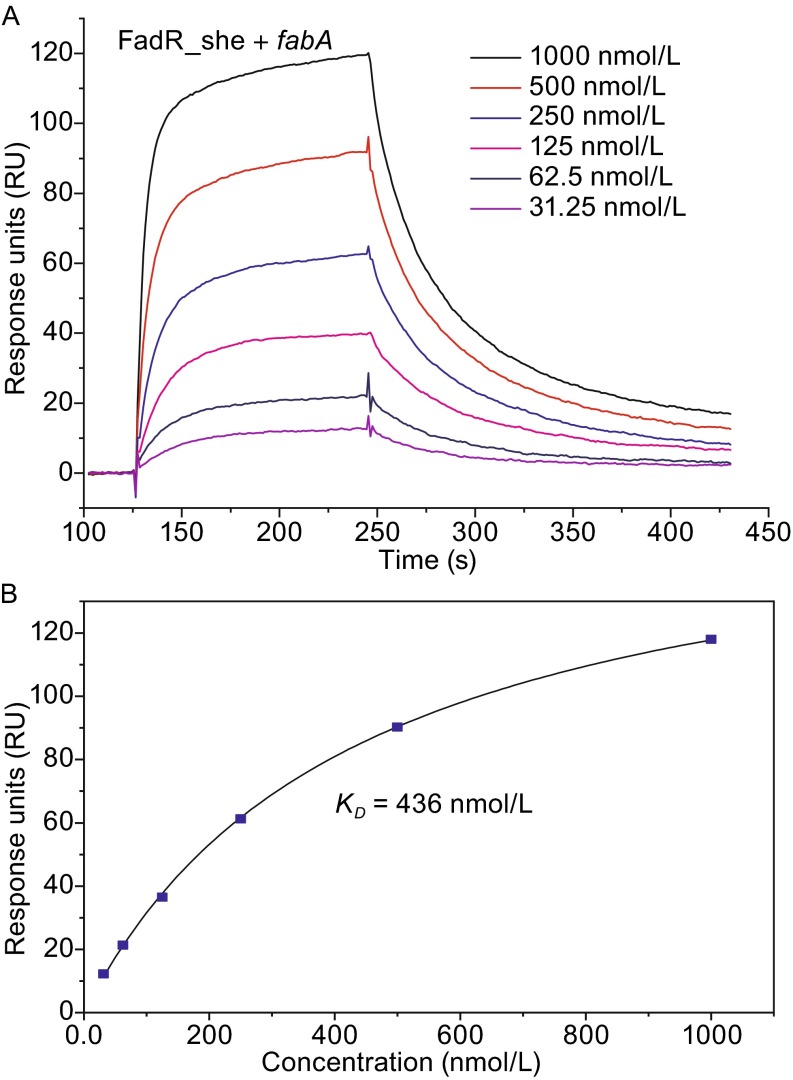
**SPR-based dynamic analyses for binding of** ***fabA*** **to** ***Shewanella*** **FadR**. (A) SPR assay for interaction between *fabA* and FadR_she. (B) Measurement of the KD value for *fabA*-FadR_she

Through sequence comparison of the *fabA* and *fabB* promoter regions of *E. coli*, *V. cholerae* and *S. oneidensis*, we found that the cognate FadR-specific binding site in front of the *fabA* promoter regions of these three bacterial species are much more conservative (Fig. S1A), but that of *fabB* promoter region is not (Fig. S1B). This observation is generally consistent with the prediction by Rodionov and coworkers (Rodionov et al., 2011) that only *fab*A (not *fabB*) of *Shewanella* has a binding site for the FadR regulator. To further prove the function of this predicted site, termed *fabA* probe, we synthesized it using the approach of annealing the two complementary DNA strand. This DNA probe is digoxigenin-labeled DNA fragment of 56 bp that overlaps the candidate FadR_she binding site (Fig. S1A and Table 2). Gel shift assays confirmed that FadR_ec (Fig. 4A) and FadR_she (Fig. 4B) both can efficiently bind the *S. oneidensisfabA* promoter. In much similarity to the scenario seen with FadR_ec here (Fig. 4A), plus our former observations with FadR regulators of *E. coli* (Feng & Cronan, 2009b, Feng & Cronan, 2010) and *V. cholerae* (Feng & Cronan, 2011b), we also found that the *fabA*_she probe binds FadR_she protein in a dose-dependent manner (Fig. 4B). To preliminarily elucidate the kinetics of FadR_she/*fabA*_she interaction, we conducted surface plasom resonance (SPR)-based measurements. SPR results revealed that the binding affinity (*K*_*D*_) of fabA_she to FadR_she is roughly 436 nmol/L (Fig. 5A and 5B), and the binding mode is 2:1 (a dimeric FadR protein is bound to a target DNA fragment (not shown).

**Figure 6:**
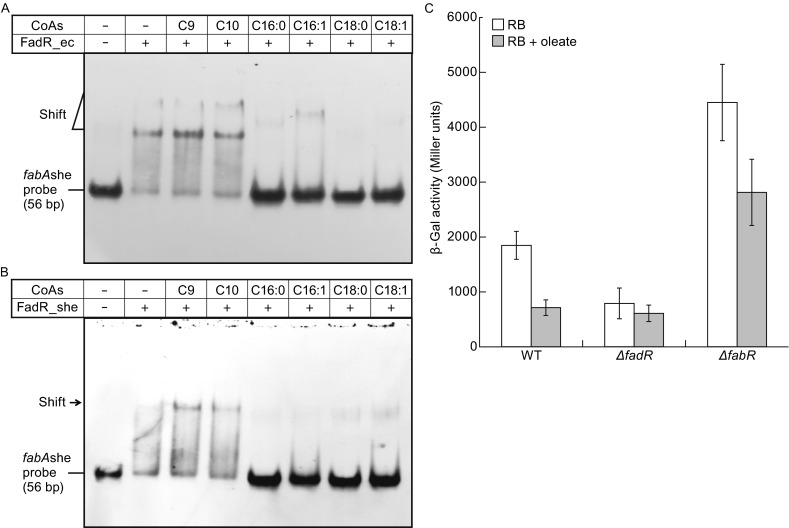
**Role of LC fatty acyl-CoA in** ***fabA*** **expression** ***in vitro*** **and** ***in vivo***. (A) EMSA-based visualization for effects of medium and long chain acyl-CoA species on binding of FadR_ec to the *fabA*she probe. **(**B) Effects of different long chain acyl-CoA species on binding of FadR_she to the *fabA*she probe. In the binding reaction mixtures (10 µL in total), the FadR (~5 pmol) was incubated with 0.2 pmol of DIG-labeled *fabA*she probe. When required, acyl-CoA (~50 pmol) was added as we recently described (Feng & Cronan, 2011b). The gel shift assays were conducted for more than three times using 7% native PAGE, and the representative result is given. The shifted *fabA*she probe band is indicated with a triangle (A) or an arrow (B). Designations C9:0, nonanoyl-CoA; C10:0, decanoyl-CoA; C16:0, palmitoyl-CoA; C18:0, stearoyl-CoA; C18:1, oleoyl-CoA. Abbreviations: ec and she denote *E. coli* and *Shewanella*, respectively. Plus sign denotes addition of either FadR proteins or acyl-CoA species, whereas minus sign denotes no addition of either FadR protein or acyl-CoA species. (C) Transcription of *fabA*she is activated by FadR in *E. coli* and repressed upon oleic acid supplementation. All the *E. coli* strains used here carried a single copy of *fabA*she*-lacZ* transcriptional fusion which is integrated on chromosomes. Bacterial cultures in mid-log phase were collected for assaying the LacZ (β-gal) activity. The three strains used here included FYJ241 (WT), FYJ246 (Δ*fadR*), and FYJ247 (Δ*fabR*), respectively. As anticipated, transcription of *fabA* she is also negatively regulated by FabR in *E. coli*

Given the fact that long-chain (but not short chain) fatty acyl-CoA species can antagonize the DNA-binding activity of FadR with origins of *E. coli* (Henry & Cronan, 1992, Cronan, 1997) and *Vibrio* (Iram & Cronan, 2005, Feng & Cronan, 2011b), it is of much interest to test the behaviors of theses ligands in the case of *S. oneidensis* FadR. Therefore, we tested six acyl-CoA species of different acyl chain lengths. The EMSA-based competition assays showed that medium-chain acyl-CoA (C9:0; C10:0) don’t interfere with the *fab*A_she binding to either FadR_ec (Fig. 6A) or FadR_she (Fig. 6B). In contrast, the long-chain acyl-CoA species (C16:0, C16:1, C18: 0 and C18:1) strongly impaired DNA binding (Fig. 6A and 6B). We believed that long-chain but not medium-chain acyl-CoA can specifically interact with FadR_she ligand-binding site and release FadR_she from the *S. oneidensisfabA* promoter. In summary, the *in vitro* data accumulated suggest that long-chain acyl-CoAs regulate the *Shewanella fabA* transcription via their interaction with the FadR protein.

### Expression of *Shewanella fab*A is activated by FadR, but repressed by oleate in *E. coli*

It is well known that: 1) FadR acts as an activator for expression of *fabA* and *fabB*, the two genes required for *E. coli* UFA synthesis, 2) whereas FabR is a repressor for expression of *fabA* and *fabB*. We construct three *E. coli* strains including FYJ241 (WT), FYJ246 (△*fadR*) and FYJ247 (△*fabR*). The three strains all carried a single copy of *fabA*she*-lacZ* transcriptional fusion on chromosomes which allows us to detect whether the *E. coli* counterpart of FadR regulatory proteins has the *in vivo* role in modulating the *ShewanellafabA* expression and to monitor the physiological effect on *fabA*_she transcription exerted by exogenous fatty acids. Measurement of the β-Gal levels of these strains showed that deletion of *fadR* eliminate its activation to *fabA*_she expression (Fig. 6C). In comparison with the wild-type strain, appreciable lower β-gal activity was seen in the △*fadR* mutant (Fig. 6C). In contrast, the removal of the opposite regulator, FabR repressor, gave significant increment of *fabA*_she expression (Fig. 6C). As expected, the activity of *fabA*_she promoter is inhibited by the addition of oleate and this down-regulation is dependent on the presence of FadR regulator (Fig. 6C). Thus, our results represent *in vivo* evidence that expression of *Shewanella fab*A is activated by FadR, but repressed by oleate.

## CONCLUSIONS

The data reported here defined that the *Shewanella* FadR homologue is a functional regulator with the involvement of fatty acid metabolism. Also, we experimentally proved the proposal by Rodionov et al. (Rodionov et al., 2011) that *fad* regulon is contracted in *Shewanella*. Retrospectively, Wang et al. (Wang et al., 2009) reported the pilot exploration of fatty acid metabolism in *Shewanella piezotolerans* with concentration on its relevance to different temperatures and pressures.

Very recently, Gao’s research group also provided genetic evidence in aiming to pose its role of *fadR* into the UFA pathway in *S. oneidensis* MR-1 (Luo et al., 2014). Right now, the picture of *Shewanella* FadR seemed to be much more complete in the context of lipid metabolism. Unlike the paradigm *E. coli* in which both *fabA* and *fabB* have the FadR-binding sites, *Shewanella* only rendered the *fabA* fatty acid synthesis gene under the control by the FadR activator (Figs. 1 and 6C) and the FabR repressor (Fig. 6C). Moreover, it is in rational to functionally assign this unparalleled regulation to its unique evolutional selection and even adaptation to its growing (/neighboring) environmental/ecological niches with the availability of limited fatty acids. Taken together, we provided integrative evidence that *Shewanella* FadR binds to the *fabA* fatty acid biosynthetic gene, which is implicated into contraction of the *fad* regulon.

## MATERIALS AND METHODS

### Bacterial strains and growth conditions

With an exception of *Shewanella oneidensis* MR-1 (*S. oneidensis*), all of the bacterial strains used here were *E. coli* K-12 derivatives (Table 1). The media are separately LB medium (10 g of tryptone, 5 g of yeast extract and 10 g of NaCl per liter), and rich broth (RBO medium;10 g of tryptone, 1 g of yeast extract and 5 g of NaCl per liter). To measure the activity of β-galactosidase in induction assays, oleate was solubilized with Tergitol NP-40 and used at a 5 mmol/L final concentration. Antibiotics were supplemented as follows (in mg/L): sodium ampicillin, 100; kanamycin sulfate, 50; tetracycline HCl, 15; and chloramphenicol, 20.

**Table 1:** Strains and plasmids used in this study

Bacteria or plasmids	Relevant characteristics			Sources
Bacterial strains				
* Shewanella oneidensis* MR-1	A Gram-negative anaerobic bacteria which is predominantly found in deep sea anaerobic habitats			Heidelberg et al. (2002), CGSC^a^
BL21(DE3)	An expression host for recombinant plasmids			Lab stock
MFH8	UB1005, Δ*fadR::*Tn*10*			Henry & Cronan (1992)
SI91	UB1005, Δ*fabR::*Cm			Feng & Cronan (2009b)
FYJ187	MC4100/pINTts			Feng & Cronan (2011b)
FYJ189	BL21 carrying pET28a-*fabR*she			This work
FYJ214	BL21 carrying pET28a-*fadR*she			This work
FYJ193	DH5α (*λ-pir*)			Lab stock, (Feng & Cronan, 2009a, Feng & Cronan, 2011b)
FYJ236	DH5α (*λ-pir*) carrying pAH-P*fabA*she			This work
FYJ241	MC4100 with a single copy of *fabA*she*-lacZ* fusion integrated at the λ-site			This work
FYJ246	FYJ241, Δ*fadR* : Tn*10*			FYJ241/*P1*_vir_(MFH8)
FYJ247	FYJ241, Δ*fabR* : Cm			FYJ241/*P1*_vir_(SI91)
Plasmids				
pET28(a)	T7 promoter-driven expression vector, Kan^R^			Novagen
pAH125	Promoter-less *lacZ* reporter plasmid in *E. coli*, Kan^R^			Feng & Cronan (2009a), Haldimann & Wanner (2001)
pET28-*fadR*ec	Recombinant plasmid carrying *E. coli fadR*, Kan^R^			Cherepanov & Wackernagel (1995), Feng & Cronan (2009b), Feng & Cronan (2009a)
pET16-*fadR*vc	Recombinant plasmid carrying *V. cholerae fadR*, Kan^R^			Feng & Cronan (2011b), Iram & Cronan (2005)
pET28-*fadR*she	Recombinant plasmid carrying *Shewalle fadR*, Kan^R^			This work
pAH-P*fabA*she	Recombinant plasmid carrying *Shewalle fabA* promoter region, Kan^R^			This work

^a^CGSC denotes Coli Genetic Stock Center, Yale University

### Plasmids and DNA manipulations

The *fabA* promoter region of *S. oneidensis* was PCR amplified and directly cloned into pAH125, giving the recombinant plasmid pAH125-P*fabA*she (Table 1). Similarly, the *fadR* gene amplified from *S. oneidensis* was inserted into expression vector pET28(a), generating the chimeric plasmid pET28-*fadR*she (Table 2). To prepare three different versions of FadR proteins, the corresponding expression plasmids (pET28-*fadR*ec, pET28-*fadR*she and pET16-*fadR*vc, in Table 2) were transformed into the strain BL21(DE3) (Feng & Cronan, 2009b). The acquired plasmids were verified by direct DNA sequencing.

**Table 2:** Primers used in this study

Primers	Primer sequences
*fadR*_she-F	5′-CG GGATCC ATG ATT ATC AAT GCC AAA GGA CC-3′
*fadR*_she-R	5′-CCG CTCGAG CTA ATG GGA GTC CTG CTG TG-3′
*fabR*_she-F	5′-CG GGATCC ATG GGT ATT CGT GCA CAG CA-3′
*fabR*_she-R	5′-CCG CTCGAG CTA CCG ATG TTC AAC TTT ATG T-3′
*fabA*_she-BD-F	5′-GAC ATT AAT T**AG CTG ATC GGA GTT GTT** T***AG CTT ACA CGT GTT CGC T***AA TCT TGG CG-3′
*fabA*_she-BD-R	5′-CGC CAA GAT T***AG CGA ACA CGT GTA AGC T***A**A ACA ACT CCG ATC AGC T**AA TTA ATG TC-3′
*fabA*_ec-BD-F	5′-TTT ATT CCG **AAC TGA** **TCG GAC** **TTG TT**C ***AGC GTA*** ***CAC GTG TTA GCT*** ATC CTG CGT GC-3′
*fabA*_ec-BD-R	5′- GCA CGC AGG AT***A GCT AAC ACG TGT*** ***ACG*** ***CT***G **AAC** **AAG TCC GAT CAG TT**C GGA ATA AA-3′
*fabB*_ec-BD-F	5′-TCT ATT AAA T**GG** **CTG** **ATC GGA CTT GTT** C***GG CGT ACA AGT GTA CGC T***AT TGT GCA TTC-3′
*fabB*_ec-BD-F	5′-GAA TGC ACA AT***A GCG*** ***TAC ACT TGT ACG*** ***CC***G **AAC** **AAG TCC GAT CAG** **CC**A TTT AAT AGA -3′
*PfabA*she-F	5′-CCG GTCGAC GAG GGT TAA CGG GTA AAC AAG-3′
*PfabA*she-R	5′-AACC GAATTC GTC GAT CAT CAG CAT GTT GTC-3′
*LacZ-R*	5′-GAC CAT GAT TAC GGA TTC ACT G-3′

The sequences underlined are restriction sites. Putative FadR-binding sites are in bold letters, and the predicted FabR palindromes are indicated in bold and italic

Given that the replication of pAH-P*fabA*she plasmid requires the presence of *pir* protein, it thus was maintained in strain DH5α *λ*-*pir* (Table 1). To impart antibiotic resistance in *E. coli* MC4100 (a *lacZ* strain lacking *pir*), this plasmid must specifically integrate into the *attλ* site of bacterial chromosome in a reaction catalyzed by the pINT-ts helper plasmid, giving strain FYJ241 carrying *fabA*she-*lacZ* transcriptional fusion (Table 1). PCR assay was applied to confirm the *fabA*she-*lacZ* junction.

### *P1*vir phage transductions

*P1*vir transductions were conducted as described by Miller (Miller, 1992) with little changes. The strain FYJ246 was constructed by P1vir transduction of strain FYJ241 using a lysate grown on strain MFH8 (Δ*fadR*::Tn*10*) with selection for tetracycline resistance. Similarly, strain FYJ241 was transduced by *P1*vir lysate obtained from strain SI91 (Δ*fabR::*Cm) with selection for kanamycin, giving strain FYJ247 (Table 1). The relevant genotypes of the acquired strains were proved by PCR analyses.

### β-Galactosidase assays

Mid-log phase cultures grown in either LB or RB were collected by spinning, washed with Z-buffer and suspended in Z-buffer for further measurement of β-galactosidase activity (Feng & Cronan, 2009b, Feng & Cronan, 2009a, Miller, 1992). The data were recorded in triplicate in no less than three independent assays.

### Expression and purification of three different FadR proteins

In addition to the FadR protein with origins of both *E. coli* and *V. cholerae*, the *S. oneidensis* FadR protein was produced in solubility via the induced expression with 0.2 mmol/L isopropyl β-D-1-thiogalactopyranoside (IPTG) at 30°C for 3.5 h. The bacterial lysis by two rounds of sonication treatment was clarified by centrifugation, and the resultant supernatant was loaded onto a nickel-ion affinity column (Qiagen). The contaminant proteins were removed with wash buffer containing 50 mmol/L imidazole, and subsequently the 6× His-tagged FadR proteins in three versions (FadR_she, FadR_ec and FadR_vc) were eluted in elution buffer containing 100 mmol/L imidazole. The protein was concentrated by ultra-filtration (30 kDa cutoff) and exchanged into 1× PBS buffer (pH 7.4) containing 10% glycerol. The purified proteins were visualized by 15% SDS-PAGE followed by staining with Coomassie Brilliant Blue R250 (Sigma, St. Louis, MO).

### Size exclusion chromatography

Given the fact that both FadR_ec and FadR_vc can form dimer, we aimed to check the solution structure of FadR_she. Therefore, we used a Superdex 75 column (Pharmacia) run on an Äkta fast protein liquid chromatography system (GE Healthcare) (Feng & Cronan, 2010, Feng & Cronan, 2011a) to perform gel filtration analyses for the purified FadR_she protein. In our trials, the column effluent was monitored at a flow rate of 0.35 mL/min in running buffer (20 mmol/L Tris-HCl, 50 mmol/L NaCl, pH 7.9). The peaks of interest were collected and confirmed with 15% SDS-PAGE.

### Liquid chromatography quadrupole time-of-flight mass spectrometry

The identity of the recombinant FadR_she protein we produced was confirmed using A Waters Q-Tof API-US Quad-ToF mass spectrometer connected to a Waters nano Acquity UPLC) (Feng & Cronan, 2011a). In brief, the protein band of interest was cut from 15% SDS-PAGE gel, de-stained and digested with Sequencing Grade Trypsin (G-Biosciences St. Louis, MO, 12.5 ng/μL in 25 mmol/L ammonium bicarbonate); Second, the resulting peptides were loaded on a Waters Atlantis C-18 column (0.03 mm particle, 0.075 mm × 150 mm), following the further cleaning treatment. The data dependent acquisition combined with ms/ms analysis was routinely performed (Feng & Cronan, 2011a).

### Chemical cross-linking assays

To further test the solution structure of *S. oneidensis* FadR, we carried out the experiments of chemical cross-linking with ethylene glycol bis-succinimidylsuccinate (Pierce) as we described before (Feng & Cronan, 2010). In each chemical cross-linking reaction (15 μL in total), the purified FadR protein (~10 mg/mL) was separately mixed with cross-linker at different concentrations (0, 0.1, 0.2, 0.5, 1.0, 1.5 and 2.0 mmol/L), and kept 30 min at room temperature before analysis. Note: the only protein without EGS addition serves as the negative control. All the reaction products were detected using 15% SDS-PAGE.

### Electrophoretic mobility shift assays

To document the function of FadR-binding site located in the *S.oneidensisfabA* promoter, gel shift assays were performed as we earlier described (Feng & Cronan, 2009b, Feng & Cronan, 2010, Feng & Cronan, 2011a) with minor modifications. Totally, three sets of DNA probes (Table 2) corresponded to *fabA*ec, *fabB*ec and *fabA*she, respectively. They were all generated by annealing two complementary oligonucleotides (e.g., *fabA*_she-BD-F plus *fabA*_she-BD-R, in Table 2) through the incubation at 95°C in TEN buffer (10 mmol/L Tris-HCl, 1 mmol/L EDTA, 100 mmol/L NaCl, pH 8.0) for 5 min followed by slow cooling to 25°C. The digoxigenin-labeled DNA probes (~0.2 pmol) were mixed with purified FadR (in appropriate concentrations) in the binding buffer (Roche) and incubated 20 min at room temperature. The DNA/protein mixtures were then analyzed by the native 7% PAGE, and directly transferred onto nylon membrane by contact blotting-aided gel transfer. Following appropriate treatments, the chemical-luminescence signals were captured by an exposure of the membrane to ECL films (Amersham).

### Surface plasmon resonance

Biacore3000 instrument (GE Healthcare) was utilized to carry out the surface plasmon resonance (SPR)-based measurement. The biotinylated *fabA*_she DNA probe was immobilized by streptavidin on the chip surface. The SPR assay was run in the running buffer (20 mmol/L Tris-HCl, pH 7.5, 200 mmol/L NaCl and 0.005% Tween 20) at the flow rate of 30 μL/min. FadR_she protein in a series of dilution was injected and passed over the chip surface for 2 min. Kinetic parameters were analyzed using a global data analysis program (BIA evaluation software), and final graph was given with the Origin software.

### Bioinformatic analyses

The amino acid sequences of FadR regulator are derived from *E. coli*, *V. cholerae* and *S. oneidensis* MR-1. The FadR-binding sites were all sampled from RegPrecise database (http://regprecise.lbl.gov/RegPrecise/regulon.jsp?). The multiple alignments were conducted using the program of ClustalW2 (http://www.ebi.ac.uk/Tools/clustalw2/index.html), and the resultant output was further processed by the program ESPript 2.2 (http://espript.ibcp.fr/ESPript/cgi-bin/ESPript.cgi), giving the final version of BLAST photography.

## Electronic supplementary material

Supplementary material 1 (PDF 358 kb)

## ABBREVIATIONS

FAS: fatty acid synthesis
FPLC: fast protein liquid chromatography
IPTG: isopropyl β-D-1-thiogalactopyranoside
LCFA: long-chain fatty acid
UFA: unsaturated fatty acid
