# Supplementary material for: Binding of Shewanella FadR to the fabA fatty acid biosynthetic gene: implications for contraction of the fad regulon
Source: Protein Cell. 2015 Jun 7;6(9):667–79. doi: 10.1007/s13238-015-0172-2 (PMC4537474; doi:10.1007/s13238-015-0172-2)
Supplement: Supplementary file 1 — Supplementary material 1 (PDF 358 kb) [file 13238_2015_172_MOESM1_ESM.pdf]

Supplementary figures

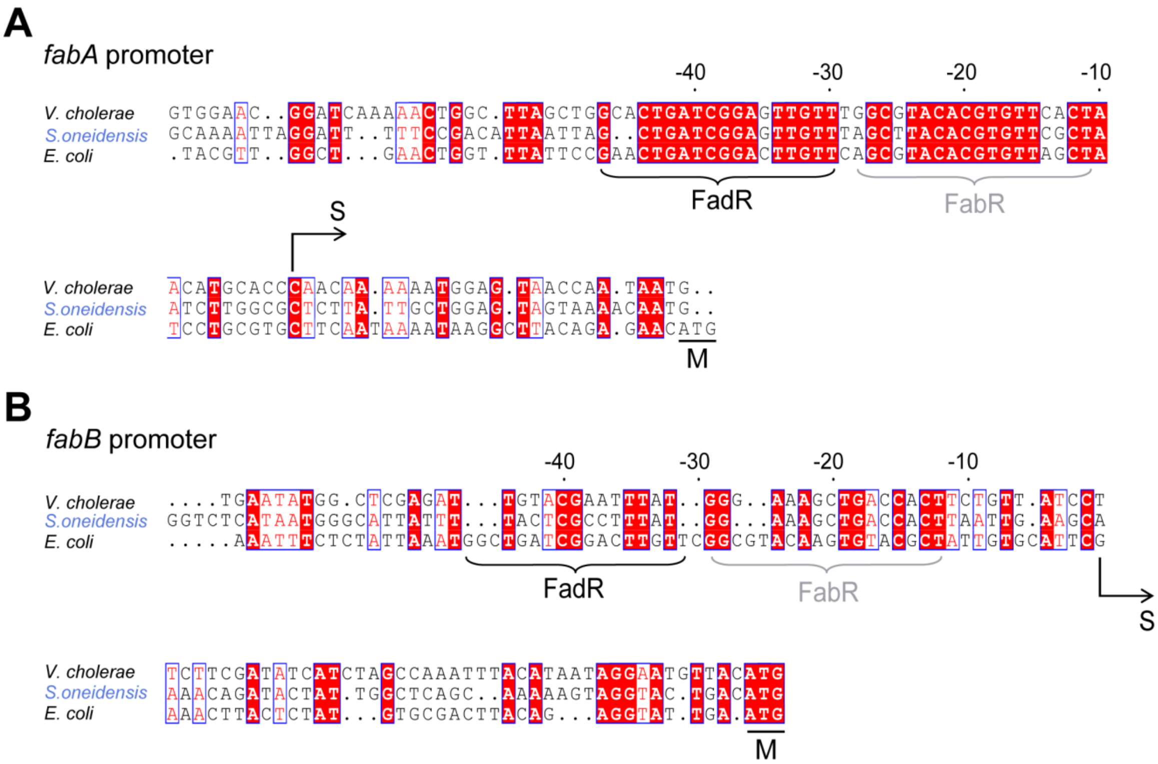

Fig. S1 Molecular dissection for promoters of *fabA* and *fabB*, the two genes for UFA synthesis

A. Sequence analyses of the *fabA* promoter

B. Sequence analyses of the *fabB* promoter and *fabB*, the two genes for UFA

The multiple sequence alignments of promoter sequences was conducted using ClustalW2 (http://www.ebi.ac.uk/Tools/clustalw2/index.html), and the resultant output was further processed with the program ESPript 2.2 (http://espript.ibcp.fr/ESPript/cgi-bin/ESPript.cgi) (Feng & Cronan, 2011b). Identical residues are in white letters with red background, similar residues are in red with white background, the varied residues are in black letters, and gaps are denoted with dots.

The FadR sites are highlighted with the black bracket, whereas the FabR sites are indicated with grey bracket. Abbreviations: S, Transcriptional start site; M: Translational initiation site.
